# Reversible changes in the 3D collagen fibril architecture during cyclic loading of healthy and degraded cartilage

**DOI:** 10.1016/j.actbio.2021.09.037

**Published:** 2021-12

**Authors:** Sheetal R Inamdar, Sylvain Prévost, Nicholas J Terrill, Martin M Knight, Himadri S Gupta

**Affiliations:** a Institute of Bioengineering and School of Engineering and Material Science, Queen Mary University of London, London E1 4NS, UK; b Beamline ID02, European Synchrotron Radiation Facility, Grenoble, France; c Harwell Science and Innovation Campus, Diamond Light Source, Harwell, Didcot OX11 10 0DE, UK

**Keywords:** Cartilage, Collagen fibrillar nanomechanics, Extracellular matrix, Synchrotron small-angle x-ray scattering, Inflammation, Osteoarthritis

## Abstract

Biomechanical changes to the collagen fibrillar architecture in articular cartilage are believed to play a crucial role in enabling normal joint function. However, experimentally there is little quantitative knowledge about the structural response of the Type II collagen fibrils in cartilage to cyclic loading *in situ*, and the mechanisms that drive the ability of cartilage to withstand long-term repetitive loading. Here we utilize synchrotron small-angle X-ray scattering (SAXS) combined with *in-situ* cyclic loading of bovine articular cartilage explants to measure the fibrillar response in deep zone articular cartilage, in terms of orientation, fibrillar strain and inter-fibrillar variability in healthy cartilage and cartilage degraded by exposure to the pro-inflammatory cytokine IL-1β. We demonstrate that under repeated cyclic loading the fibrils reversibly change the width of the fibrillar orientation distribution whilst maintaining a largely consistent average direction of orientation. Specifically, the effect on the fibrillar network is a 3-dimensional conical orientation broadening around the normal to the joint surface, inferred by 3D reconstruction of X-ray scattering peak intensity distributions from the 2D pattern. Further, at the intrafibrillar level, this effect is coupled with reversible reduction in fibrillar pre-strain under compression, alongside increase in the variability of fibrillar pre-strain. In IL-1β degraded cartilage, the collagen rearrangement under cyclic loading is disrupted and associated with reduced tissue stiffness. These finding have implications as to how changes in local collagen nanomechanics might drive disease progression or vice versa in conditions such as osteoarthritis and provides a pathway to a mechanistic understanding of such diseases.

**Statement of significance:**

Structural deterioration in biomechanically loaded musculoskeletal organs, e.g., joint osteoarthritis and back pain, are linked to breakdown and changes in their collagen-rich cartilaginous tissue matrix. A critical component enabling cartilage biomechanics is the ultrastructural collagen fibrillar network in cartilage. However, experimental probes of the dynamic structural response of cartilage collagen to biomechanical loads are limited. Here, we use X-ray scattering during cyclic loading (as during walking) on joint tissue to show that cartilage fibrils resist loading by a reversible, three-dimensional orientation broadening and disordering mechanism at the molecular level, and that inflammation reduces this functionality. Our results will help understand how changes to small-scale tissue mechanisms are linked to ageing and osteoarthritic progression, and development of biomaterials for joint replacements.

## Graphical abstract

**Figure:**
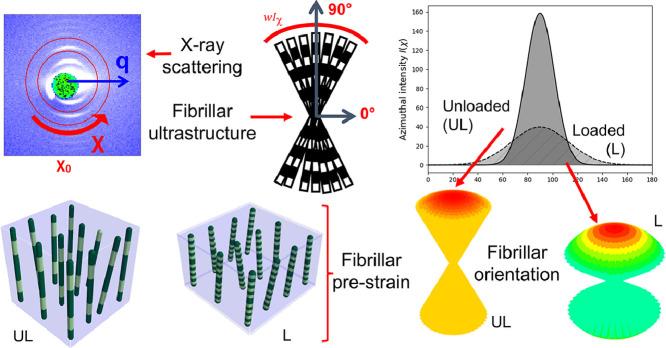

## 1 Introduction

Collagen type II fibrils form part of the structural meshwork of the extracellular matrix (ECM) in articular cartilage (AC) [1], [2], [3], [4]. Together with the proteoglycans (PG), the fibrils form a fibre-composite type network providing the tissue with structural integrity whilst playing an active role in enabling cartilage to withstand high compressive loads that are experienced during normal activity [2,[5], [6], [7]]. This fibre-reinforced tissue behaves in a poroelastic manner, which is driven by the collagen network resisting the swelling of the PG and associated water and thus leads to a pre-strained state in the fibrils [8], [9], [10]. Recently, application of X-ray and nonlinear microscopy studies have begun to shed light on the mechanics of the fibrillar network in cartilage [11], [12], [13], [14]. The active role played by fibrils in response to tissue loading was demonstrated [12] through recoverable changes in fibrillar strain as measured via the axial periodicity (D-period∼65–67 nm) in collagen fibril density under stress relaxation. On compression, the fluid flow out of the interfibrillar space is likely to cause a relative change in the strain within the fibrils. Further, it has been highlighted recently that the fibrils are sensitive to compositional changes in the ECM (and thus degradative conditions as in ageing or disease) [11,12]. These changes in fibril-level pre-strain are coupled with disordering of the intra-fibrillar structure, as measured under statically loaded conditions [11,12]. Previous studies have utilised quantitative microscopy methods to investigate the influence of dynamic loading on the individual zones of AC at the microscale and indicated that the superficial and transitional zones experience a higher level of deformation when compared to the deeper zone under repetitive loading [15]. The collagen varies both in orientation and concentration across the tissue [16] and therefore it is essential to understand the contribution of the fibrillar level network in resisting deformation in the different zones. Mansfield et al have recently investigated the organisation and molecular ordering of the collagen fibrils in AC under tensile loading using polarization sensitive second harmonic generation (P-SHG), finding that at the sub-micron scale the fibrils re-organised in relation to the applied strain, whereby the orientation shifts towards the direction of the strain [14]. These recent studies all highlight the importance of the collagen network in the response to loading. However, little is known experimentally about collagen fibril deformation *in situ* under dynamic repetitive loading, as in locomotion [17], are very limited.

The nature of such structural response at multiple hierarchical levels in the ECM, and the contribution of specific biomolecular components therein, have implications for tissue development and pathogenic alterations. Kaab *et al* [18] showed experimentally that there was significantly higher deformation of the collagen network found in all the zones of AC when placed under static loading when compared to cyclically loaded joints however fibrillar reorientation was present in both loading conditions. Alongside this, at the tissue level, recovery to load-induced deformation appeared to be faster in a cyclically loaded case [18]. A more recent study by Nia *et al* [19] combined an oscillatory loading study with a fibril-reinforced poroelastic model which found that poroelasticity was the driving factor in the frequency dependent mechanical behaviour found in cartilage. The loading regime significantly affects both the structural response between the individual tissue zones of AC as well as the modulation of ECM turnover [20], [21], [22]. Through the zonally-variable localised strain response, a change in the localised collagen organisation directly impacts the mechanical forces experienced by the cells through the pericellular matrix [14,[23], [24], [25], [26]]. Numerous studies have investigated the relationship between tissue loading and the concurrent metabolism of the ECM in AC experimentally and in combination with modelling applications [17,25,27]. Thibault *et al* [27] showed that under cyclic loading, the collagen network becomes weakened and partially degraded, as indicated by a reduction in the tensile stiffness and increase in the presence of denatured collagen II measured directly after loading via an immunoassay [28], which in turn leads to an increase in the hydraulic permeability of the tissue. Such load-driven changes to the ECM directly cause an alteration in the localised environment of the cells. The study also found that explants maintained in culture post-loading no longer expressed an increase in denatured collagen, which suggests some level of turnover or repair in the ECM [27]. As such, these load-driven events in a healthy condition would occur in a balanced state, while instability in the transduction of load via the ECM may contribute to the pathogenesis of diseases such as osteoarthritis (OA) [24,26,29].

In such degradative states such as OA, the disruption to the load induced turnover of the ECM is thought to involve an inflammatory response [30,31]. Pro-inflammatory cytokines such as interleukin-1β (IL-1β), are secreted by both the chondrocytes and the synoviocytes found in the synovial sublining layer [32,33]. Elevated levels of IL-1β have been reported in humans with osteoarthritis at concentrations between 0.021 to 0.146 ng/ml compared to levels up to 0.020 ng/ml in healthy subjects [34]. IL-1β leads to increased production of nitric oxide (NO) and prostaglandin E_2_ (PGE_2_) both of which are inflammatory mediators leading to the upregulation of matrix metalloproteases (MMPs) [35], such as MMP-13 which primarily drives collagen type II cleavage [36], and subsequent cartilage degradation. In the longer term IL-1β causes suppression in collagen synthesis [37]. As a result of these molecular changes, there is a direct impact on the tissues mechanical integrity reducing the stiffness of cartilage, and the load bearing capacity [25]. In OA, this further leads to surface fibrillations and osteoarthritic lesions which over time develop to complete tissue breakdown, when combined with continued physiological loading [38]. In terms of the tissue level mechanics, a study by Thompson *et al.* (2015) found that IL-1β treatment led to approximately a 60% reduction in the tangent modulus from 2.42 MPa in the control to 0.88 MPa in the IL-1β group. Alongside this, the relaxation modulus was also reduced by approximately 75% whilst the relaxation half-life was also significantly shorter in the IL-1β group indicating alterations in both the solid elastic and poroelastic behaviour of fluid movement [38]. IL-1β treatment is therefore a well-established method to induce cartilage matrix degradation in a similar manner to that which occurs *in vivo* during inflammatory joint disease such as osteoarthritis [39]. However, there is little understanding into how the mechanics of the collagen fibrillar network is impacted by such inflammatory conditions in terms of structural and functional responses.

When considering the mechanics of the fibrillar network in AC, the case of physiological cyclic loading in AC and the fibrillar response therein is less understood, though a few experimental studies have now revealed the fibrillar-level mechanisms in AC in response to static load or compositional changes [[11], [12], [13],^40^,^41^]. While poroelastic modelling of cartilage mechanics [8,42,43] have advanced to include the fibrillar component [44,45], direct experimental visualisation of fibril-level dynamics is missing. Recent studies on other tissue systems like tendons have shown *in-vivo* dynamic alterations of collagen structure at the molecular and supramolecular level and evidence of damage [46]. Cyclic loading simulates a more physiological loading condition within the tissue compared to static loading [47,48]. Here, we report the first study of the collagen dynamic response to cyclic loading, using *in-situ* high-brilliance synchrotron small angle X-ray diffraction (SAXD) to measure changes in the fibrillar ultrastructure in an animal model (bovine cartilage explants). Concurrently, we analyse the effect of IL-1β driven inflammation and degradation on the mechanical response of the collagen network.

## 2 Methods

### 2.1 Sample collection and cytokine treatment

Bovine cartilage explants were harvested from the metacarpal-phalangeal joints of freshly slaughtered adult bovine steers 18–24 months in age, obtained from a local abattoir. The entire joints were first washed and then soaked in both biological detergent and 70% ethanol before the joint surface was exposed. As the joint is intact during this preliminary washing step, there is no exposure of the internal cartilage tissue to the detergent or ethanol. Biopsy punches were used to produce 2 mm diameter full thickness explants whilst the joint surface was hydrated using phosphate buffered saline solution (PBS) (Sigma-Aldrich, Poole, UK). The fresh explants were then transferred into a 48-well plate in media (DMEM + Pen/Strep) and left to rest in an incubator for 24 h at 37 °C. The samples were incubated for 12 days in serum-free supplemented DMEM with and without IL-1β (5 ng/ml, Peprotech, UK). While 5 ng/ml is high compared to *in vivo* levels found in OA patients, we used this concentration as our group has previously shown IL-1 in this range produces an accelerated *in vitro* mechanical degradation over a period of 7 days (rather than months or years) [38]. In both groups media was changed change every 3 days. Following treatment, samples were transferred to PBS in Eppendorf tubes, snap frozen using liquid nitrogen and stored at -20 °C prior to small angle X-ray diffraction (SAXD) studies.

### 2.2 Mechanical loading and SAXD procedure

**Fig. 1:**
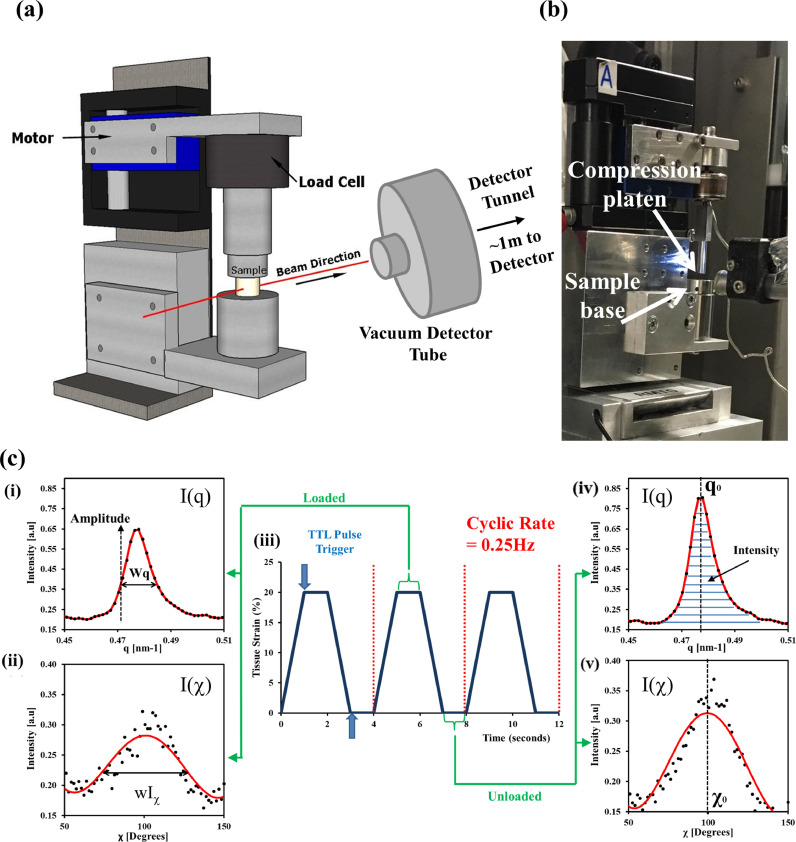
Loading/scanning protocol alongside beamline setup at ID02, ESRF. A schematic representation of the micro-compression tester is shown in (a). An image of the micro-compression tester setup at beamline ID02 at ESRF is shown in (b). The loading regime and sample integrated SAXD data is shown in (c). Part (i) shows a sample plot of I(q) integrated from a SAXD pattern taken under load application with the associated I(χ) shown in part (ii). The associated unloaded profiles are shown in (iv) and (v), respectively. Part (ii) indicates the loading profile utilised over 150 cycles with maximum tissue strain at 20% at a rate of 0.25 Hz. The large blue arrows indicate the point at where the TTL pulse trigger was sent to the detector for acquisition of a SAXD pattern and this was repeated for every 5^th^ cycle.

Experiments were conducted at the ID02 beamline [49], ESRF, using an ultra-low divergence beam with a size reduced by slits to as small as technically feasible (∼50 μm FWHM at sample and at detector) to enable spatial resolution of the measurements in the deep zone. The sample to detector distance was measured as 1006.8 ± 1.0 mm determined using a chicken collagen standard. The wavelength used was 0.095 nm with a resolution of the order of 1E-4. A FReLoN 4M CCD detector was employed, with pixel size of 23.8 µm and FWHM resolution of 44.3 ± 0.7 µm, i.e. similar to the beam size. As indicated in Fig. 1**(a)**, the detector in ID02 is in a vacuum tunnel, which significantly reduces the noise in the SAXD data associated with air scattering.

During each experiment, samples were placed within the sample holder and pre-strained to 0.1% compressive strain, whilst being hydrated with PBS, prior to any loading. Fig. 1**(b)** shows an image of the micro-compression tester setup at the ID02 beamline. An initial line scan with 50 µm increments in the y-direction (perpendicular to the tissue/joint surface) was performed to determine a zone of interest based on the qualitative differences in the SAXD patterns. Once a region was determined a specialised control-script was run to continuously move the sample-stage motors both in horizontal (x-) and vertical (y-) directions within a fixed window 500 µm wide (in x-direction) by 200 µm wide (in y-direction). The reason for the continuous scanning motion was to reduce the amount of time any part of the tissue was exposed to the beam, thus minimising radiation-damage effects. Further, to avoid excess radiation exposure, an exposure-time test was conducted to determine the minimum exposure time per point required to obtain a SAXD pattern with clear meridional peaks for analysis, which was determined to be at 0.03 sec.

The loading regime, and subsequent parameters obtained from the cyclic loading experiments are indicated in Fig. 1**(c)**. A physiologically representative strain amplitude of 20% was chosen, in line with our previous work [11,12]. A custom-build LabVIEW PC control system for *in situ* loading was used [12]. The term *in situ* here refers to the SAXS experiment, rather than the tissue, and is used when SAXS measurements are carried out during the application of the cyclic loading protocol, rather than before and after the loading. Each sample was subjected to 150 cycles at 0.25 Hz which corresponded to the lower end of the physiological loading frequency. This loading regime shown schematically in Fig. 1**(c, part iii)**, consisted of a ramp-up to 20% strain which was then held for 1 sec in the loaded state to allow for the SAXD acquisition and associated detector overhead processing times, followed by a reduction in strain to 0%, which was also maintained for 1 sec to allow a further SAXD acquisition in the unloaded state. A TTL pulse trigger, initiated from the control PC, was used to remotely control when the detector was activated. Detector acquisition was set to occur every 5 cycles in the loaded and unloaded states rather than every cycle, to reduce radiation exposure. All samples were kept in a hydrated state with phosphate buffered saline (PBS) solution, similar to our prior work [11,12]. A total of n = 4 AC cores were tested from the control group, and n = 5 from the IL1-β group.

### 2.3 SAXD analysis for fibrillar nanostructural parameters

From the SAXS patterns a combination of nano- and micro-scale parameters of cartilage ECM were measured. These reflect the changes at the (nano) fibril-level (from azimuthally integrated radial profiles *I*(q)) and at the (micro) level of fibril arrays angularly distributed in the 3D microenvironment (from radially integrated azimuthal profiles *I*(χ)); here *q* = (4π/λ) sin(2θ/2) where 2θ is the Bragg angle, and χ denote the radial and azimuthal angle coordinates in the 2D SAXS detector plane.

It is essential at the start to note that the 2D SAXS pattern captures the scattering only from those fibrils oriented at right-angles (in the detector plane with current diffraction geometry) to the X-ray beam, and fibrils out of plane will hence not be visualised in the detector signal. The 5^th^ order meridional SAXD peak was used for all fibril-level analysis of *I*(q) and *I*(χ), following the protocol used in our prior work [12], because it is both sufficiently far from the central diffuse scattering that a simple linear background correction works, and sufficiently strong to enable reliable peak fitting. On the left, Fig. 1**(c, part i)** indicates both the amplitude and width of the azimuthally integrated peak *I*(q). The width parameter w_q_ provides an indication into the level of interfibrillar molecular disorder; specifically, the variability in axial fibril D-period between fibrils in the scattering volume. On the right, the peak position q_0_ (Fig. 1**(c, part iv)**) is inversely proportional to the D-period (following Bragg's law, q_0_ = 5(2π/D), for the 5^th^ order), which serves as a measure of fibrillar pre-strain in the hydrated fibrillar/GAG cartilage nanocomposite [12]. Fig. 1**(c, part iv)** indicates the peak intensity (the area under the azimuthally integrated peak), which is proportional both to the collagen content and degree of intrafibrillar order for the in-plane fibrils. Similarly, in Fig. 1**(c, part ii)**, the angular width (wI_χ_) of the radially integrated profile *I*(χ) provides an indication into the degree of angular orientation of the in-plane fibrils satisfying the Ewald criterion (i.e. in the plane at right angles to the beam); the wider the peak, the larger range of possible fibrillar orientations in the 2D plane. The angular intensity peak position χ_0_ (Fig. 1**(c, part v))** in the radially integrated profile provides the predominant orientation of the in-plane fibrils. For clarity, Supplementary **Fig. S1** (adapted from our previous work [11,12]) shows the relation between the SAXD patterns and profiles and the fibrillar ultrastructure.

### 2.4 Statistical analysis

The variation between AC ultrastructural parameters in loaded and unloaded states was calculated in two ways:

*Time-dependent variation*: Pairwise *t*-tests of parameters like D, w_q_, 5^th^ order peak intensity I_5_, χ_0_ and wIχ in the loaded vs. unloaded states were calculated at each cycle number (*p* < 0.05 taken as significant) using Microsoft Excel.
*Averaged across all cycles*: The parameters listed above were also averaged across all cycles in the test. The averages from each sample were aggregated into loaded/unloaded groups and *t*-tests carried out to test for significant differences.

## 3 Results

### 3.1 Cyclic response of the fibrillar nanostructure in normal bovine cartilage

**Fig. 2:**
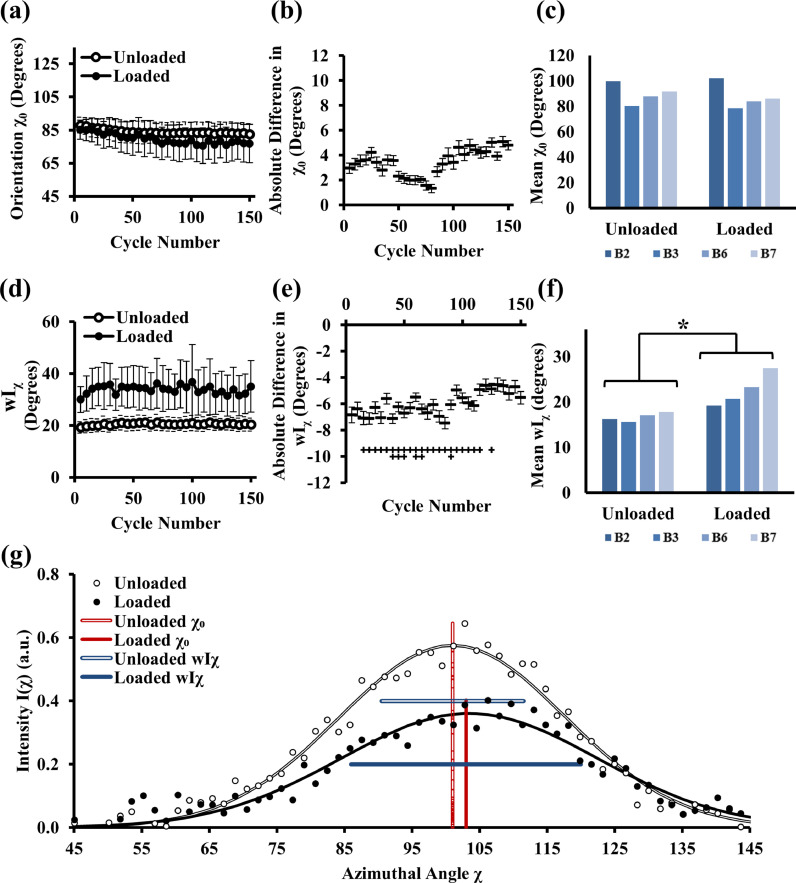
*Collagen fibrils respond to cyclic loading via an increase in angular span of orientations and subsequent partial relaxation.* The average predominant orientation of the fibrils at each measure cycle, both in the unloaded and loaded phase, is represented in (a) across 150 cycles. The pairwise difference between the orientation in the unloaded and loaded phase is indicated in (b), where there is no significant difference, as further shown in (c) where the individual sample (B2, B3, B6 and B7) mean values are plotted across the 150 cycles. The average degree of orientation (wI_χ_) in each measured cycle is shown in (d) for the unloaded and loaded phase and the total difference between these shown in (e). There is a significant difference in this parameter between unloaded and loaded states, as shown in (f) where there is a larger mean wI_χ_ under compression, indicating a broadening of the angular distribution of the fibrils. The radially integrated SAXD data is represented in part (g) for a representative cycle point. Horizontal blue lines (color online) indicate schematically the width of the peaks wI_χ_. The vertical red lines (color online) indicates the centre of the gaussian fits (fit curves shown by black lines and filled/open circles) and thus represent the direction of fibril orientation, whilst the width of the peaks indicates the broadening of the degree of orientation. Error bars represent standard error of mean throughout where n = 4, and * indicates the pairwise significance between the loaded and unloaded groups across all cycles where *p* < 0.05 (*) in part (f). The + symbol in part (e) indicates the significance of the difference between the unloaded and loaded phase at each measured cycle, where *p* < 0.05 (+) and *p* < 0.01 (++). For (e), note that the +, ++, and +++ symbols at each cycle number are written vertically due to the close horizontal spacing (this convention is retained for subsequent Fig.s).

Fig. 2**(a)** shows that cyclic loading does not cause a significant change to the mean direction of orientation of the fibrils, as there is a minimal difference between χ_0_ in the loaded and unloaded phase (∼1°–6°). While Fig. 2**(a)** averages over all samples at each loading phase, a pairwise calculation of the total difference between the two phases shows lack of a clear temporal trend or significant difference as shown in Fig. 2**(b)**. There appears to be a slight upward trend between the 100–150 cycle mark. Further, there is no significant difference between loaded and unloaded state in the average of the orientation angle (over the long term, i.e., averaged across all cycles) for each individual sample as shown in Fig. 2**(c)**.

In contrast to the minimal change in average fibril angular direction, there is a clear increase in the wI_χ_ parameter on loading (Fig. 2**(d)**), suggesting a broadening of the angular distribution of the in-plane fibrils under loading. This is a reversible effect, as the wI_χ_ parameter reverts back when unloaded to around 20°, while in the compressed state there is a range of wI_χ_ (mainly between 30°–40°) equating to almost double in angular width change. The significance of the load-induced change in wI_χ_ is further supported by the absolute-difference plot in Fig. 2**(e)**, where there is a significant difference between the two phases for most of the 150 cycles up to around 125 cycles. After ∼125 cycles there is a slight upward trend, where the load/unload difference in wI_χ_ reduces from 8° down to ∼4°(∼50% change). Similarly, the long-term mean of the unloaded- versus loaded-values also shows a significant increase on loading (*p* < 0.05), as shown in Fig. 2**(f)**, which is consistent across all samples. Fig. 2**(g)** shows the data from a representative radially-integrated SAXD plot in (g), where both the broadening of the I(χ) curve and the shift in orientation angle are visible under compression.

**Fig. 3:**
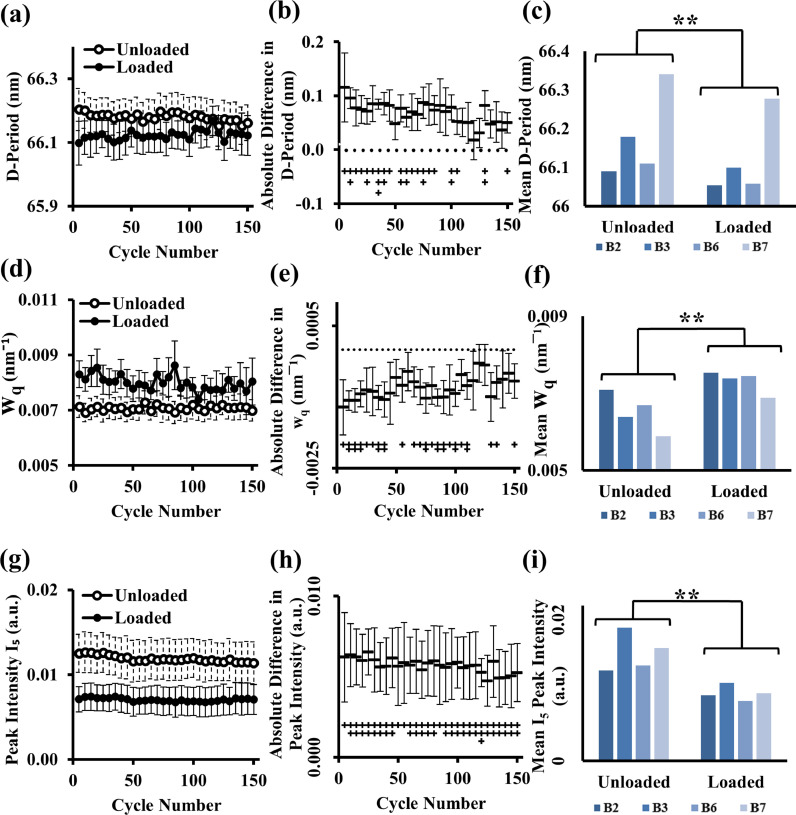
*Under cyclic loading, collagen fibrils exhibit a reversible reduction in D-period (fibrillar pre-strain), accompanied by an increase in the inter-fibrillar heterogeneity in D-period*. The long-term cyclic trend, split in to the unloaded and loaded phase of each cycle, for the D-period, w_q_ and peak intensity is shown in (a), (d) and (g), respectively whilst the total difference in these two phases is shown in (b), (e) and (h), respectively. When considering the absolute pairwise difference between loaded and unloaded state, a significant difference is observed along the trends in all parameters, with however less of a difference in the last 50 cycles for the D-period and w_q_. The mean of the individual samples (B2, B3, B6 and B7) across all 150 cycles for D-period, w_q_ and peak intensity is shown in parts (c), (f) and (i), respectively, where a significant decrease in D-period and peak intensity is observed under cyclic compression combined with a significant increase in w_q_. Error bars represent standard error of mean throughout where n = 4, and * indicates the pairwise significance between the loaded and unloaded groups, where *p* < 0.05 (*) and *p* < 0.01 (**). The + symbol in parts (b), (e) and (h) indicates the significance of the difference between the unloaded and loaded phase at each measured cycle, where *p* < 0.05 (+), *p* < 0.01 (++) and *p* < 0.001 (+++).

In a complementary manner to the angular-distribution changes in Fig. 2, the changes in axial fibrillar D-periodicity (and associated parameters), when the cartilage is placed under cyclic loading, are highlighted in Fig. 3. A very significant reduction (*p* < 0.05) in D-period is shown in Fig. 3**(a)–(c)**, where there is a change from around 66.2 nm to around 66.1 nm in the initial cycles. Whilst the D-period change appears small, it equates to a 0.3% reduction in pre-strain, falling into the same range as previously measured for fibrils under physiological levels of static compression [11]. This pre-strain difference becomes less apparent over successive cycles, as at 150 cycles the difference reduces to 0.06%, suggesting that the fibrils may be tending towards an equilibrium state where cyclic loading to specific strain levels results in minimal changes at the fibrillar level. The compression-led reduction in D-period is further demonstrated in part (b), where over most of the cycles (∼first 100), there is a significant reduction in D-period on loading, compared to unloaded state. Further, a downward trend is observed in the total difference between the two phases, corresponding to the reduced difference in the last 50 cycles referred to earlier when discussing wI_χ_. The average of the long-term trends also clearly shows the reduction in D-period (Fig. 3**(c)**), where each sample consistently reduces in D-period under compression.

Proceeding to interfibrillar variability in pre-strain or D-period (w_q_), Fig. 3**(d)** shows that there is an increase in the w_q_ parameter under compression, by around 22% in the initial cycles, which then reduces over successive cycles by approximately half to 11% by 150 cycles. The unloaded value of w_q_ remains steady over the cycle range, suggesting a stable unloaded reference state that is maintained despite repetitive loading. The difference of w_q_ between the loaded/unloaded states reduces over successive cycles, seen in the increasing trend in Fig. 3**(e)**, where the significance of the difference reduced after ∼100 cycles. This behavior is similar to that seen in the D-period and wI_χ_. As with the D-period, the long-term average trend in w_q_ is shown in Fig. 3**(f)**, which indicates an increase of w_q_ under loading across individual samples.

The total SAXD peak intensity I_5_ (Fig. 3**(g)**) reduces considerably under compression, and this behavior is consistent across all the cycles, with a reduction by around 49% which is recovered in the unloaded phase. There is a slight reduction in the intensity-difference over time, when considering the long-term trend in Fig. 3**(h)**. However, the difference between the loaded and unloaded phases remains significant across the cycle range. This intensity change is further supported by the averaged long-term trends shown in Fig. 3**(i)**, where there is a consistent, very significant (*p* < 0.01) drop in peak intensity under loading across all samples.

### 3.2 Cyclic response of the fibrillar nanostructure in bovine cartilage treated with IL-1β

In reporting the effects of IL-1β on the nanoscale response to cyclic loading, we focus here on data in which IL-1β treated samples differ from untreated controls (all other data is presented in the Supplementary Information).

**Fig. 4:**
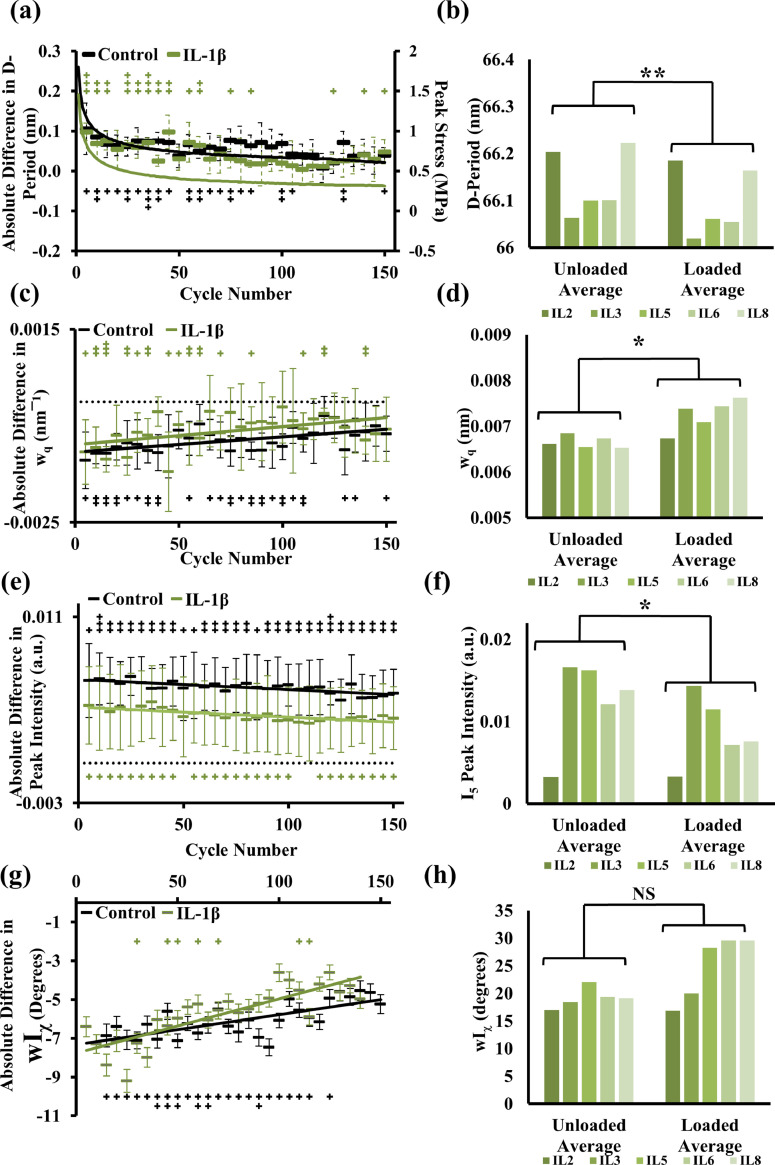
*IL-1β treatment leads to an altered mechanical response at the macroscale combined with an inability of the fibrils to significantly change their angular degree of orientation under compression*. The absolute pairwise difference between loading and unloading in each sample group for the D-period, w_q_, peak intensity and degree of orientation is shown in (a), (c), (e) and (g), respectively, where the black points (and lines) represent the control samples whilst green points and lines (color online) show the Il-1β treatment group. In part (a), the two lines represent the peak stress values at each cycle whilst in (c), (e) and (g) the two lines are linear regressions as a guide to the eye and the dashed line indicates 0 where applicable. The (+) symbols indicate the significant difference of the unloaded and loaded phase at each point where black (+) represents the control group points and green (+) represent the IL-1β group points. The average of the long-term trends for each sample within each phase for the orientation and degree of orientation is shown in (b), (d), (f) and (h), respectively. Error bars represent standard error of mean throughout where n = 5 and * indicates the pairwise significance between the loaded and unloaded groups across all cycles where *p* < 0.05 (*) and *p* < 0.01 (**). The + symbol in parts (a), (c), (e) and (g) indicates the significance of the difference between the unloaded and loaded phase at each measured cycle, where *p* < 0.05 (+) and *p* < 0.01 (++).

Cartilage explants treated with IL-1β show a greater degree of macroscale stress relaxation of peak stress values measured in the loaded phase during cyclic loading, as shown by the solid curves in Fig. 4**(a)** (see also Supplementary **Fig. S2** for group-averaged comparison with untreated controls). The peak stress values are reduced by 20% in IL-1β treated samples compared to control, with values of approximately 0.2 MPa and 0.25 MPa, respectively. However, at the fibrillar-level, when comparing the temporal variation in the total difference in D-period between the IL-1β- and control groups, the behaviour appears similar (Fig. 4**(a)**). The one main difference that can be observed is that significant differences between the loaded and unloaded phases are less frequent after around 50 cycles for the IL-1β group compared to controls, suggesting that the fibrils may reach a relaxed state faster in the treatment group, which may be linked to the initial disparity in D-period in the unloaded state. The average over all the cycles is also significantly reduced under loading, as shown in Fig. 4**(b)**.

When considering the w_q_ parameter, the trend line representing the IL-1β treatment group (as shown in green in Fig. 4**(c)**) indicates a reduced difference between the loaded and unloaded phase when compared to the control, suggesting that there is less of a range in the D-periodicity variability and therefore less inter-fibrillar heterogeneity. The time-course of the w_q_ difference still follows an upward trend like the control, with the difference reducing over successive cycles and the fibrils are tending towards an equilibrium state. As with the control, there is a significant increase in the w_q_ parameter when considering the long-term average over all cycles of each individual sample, as shown in Fig. 4**(d)**.

The *I*(q) total peak intensity changes associated with IL-1β treatment under cyclic loading are shown in Fig. 4**(e–f)**. As with the w_q_ parameter, the difference between the loaded and unloaded phases is smaller in the IL-1β group when compared to the control as indicated by the green line in Fig. 4**(e)**. This difference is also found to be less significant in the IL-1β group as indicated by the number of + signs whereby there is a larger difference between the two phases in the control group. When combined with the lower w_q_, this could indicate a higher level of disorganisation within the degraded group driven more so by structural organisation. The lowered difference in intensity could be driven by a loss in the flexibility and range of the fibrils, and similarly to the controls, the long-term average of total intensity significantly reduces under compression when considering the full cyclic range of each sample. Further, this indicates that cyclic compression causes reorganisation of the fibrils in the IL-1β states as well as controls.

The main observed difference under IL-1β treatment is found in a change to the fibrillar response in terms of angular distribution under compression. The angular range of fibrillar orientations (wI_χ_) increases less under loading, as shown in Fig. 4**(h)** where there is no significant difference. This is in contrast to the angular fibrillar range for the controls (Fig. 2), whereby, the lesser increase of the wI_χ_ parameter under compression indicates a lower increase in fibrillar angular orientation in the IL-1β group. As fibrillar rearrangement is affected by interactions between fibrils mediated by the hydrated PG-rich interfibrillar matrix, the foregoing may suggest that the IL-1β treatment leads to a lack of PG driven pre-strain amongst the fibrillar groups which could be down to the loss of PG [37] and thus associated fluid. When considering the total difference of wI_χ_ in Fig. 4**(g)**, there is a lower difference in the IL-1β group, with less significant differences across the full cyclic range (as indicated by the + signs). However, the trend remains the same as in the control case, with the range of fibrillar orientation reducing over successive cycles, again suggesting that the fibrils are tending towards an equilibrium state. Further, while wI_χ_ is larger for the loaded case (averaged across all cycles), this difference is not significant (*p* = 0.06) in contrast to the control-group (*p* = 0.02), suggesting that IL-1β treatment alters the local environment enough to prevent the fibrils from fully changing their angular distribution in response to loading.

## 4 Discussion

The response of type-II collagen fibrils in articular cartilage to cyclic loading *in situ*, in both controlled and under inflammatory conditions, has been explored in this study. The importance of the collagen fibrillar network in the response to loading has been highlighted in fibril-reinforced models however there have been limited experimental studies that support these findings [44,45]. We show here for the first time the reversibility in the structural response of the fibrils under repeated compressive loading, through changes in the inter-fibrillar organization and intra-fibrillar structure. These changes are detected via changes in fibrillar-level pre-strain, 3D fibrillar orientation and intra- and interfibrillar disorder, determined by time-dependent synchrotron SAXS with in situ loading.

**Fig. 5:**
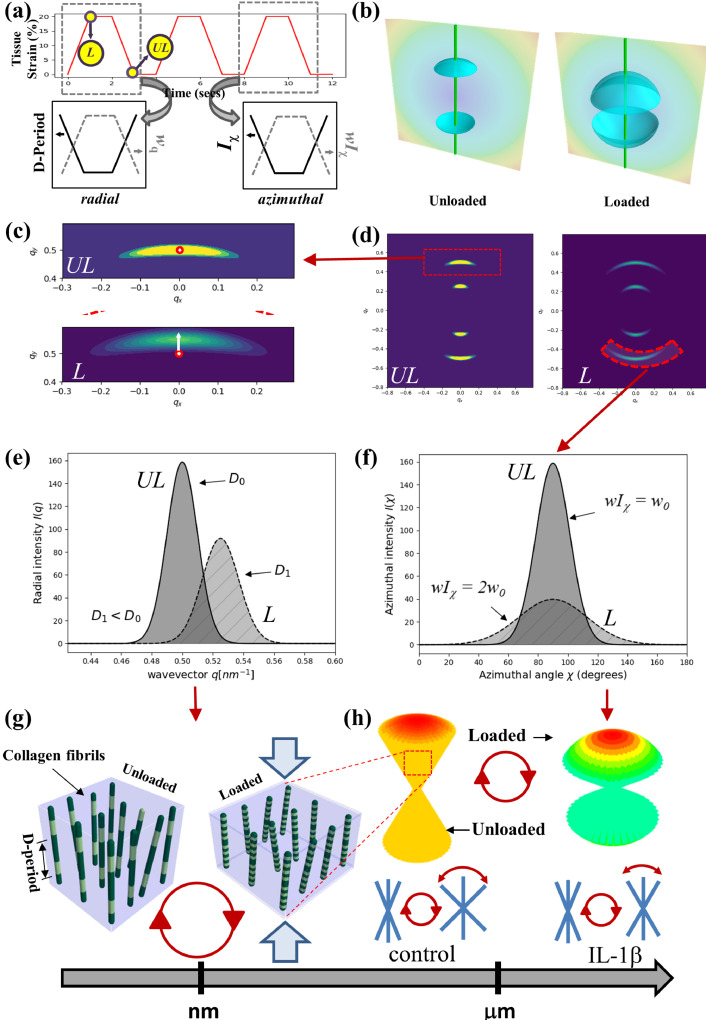
*A model for nano- and microstructural dynamics of deep-zone cartilage collagen fibrils under cyclic compression*. (a) top: Cyclic loading protocol to fixed strain at 0.25 Hz leads to (bottom: left) a decrease in fibril D-period and increase in axial peak width w_q_ and (bottom: right) a decrease in azimuthal intensity I_χ_ and increase in azimuthal peak width wI_χ_. L: loaded state; UL: unloaded state. (b) Simulations of a 3D model of SAXS scattering from cartilage collagen fibrils, with the 5^th^ order peak shown via a parametric surface plot, for an axially symmetric narrow (left) and broad (right) 3D orientation distribution, with the Ewald sphere intersection with the scattering intensity shown via the vertical curved surfaces (in the SAXS-regime, approximately flat planes). (c) and (d) show resultant 2D simulated SAXS patterns (only 3^rd^ and 5^th^ order peaks shown), demonstrating i) the azimuthal broadening and weakening of the meridional Bragg reflections ((d), right: dashed arc) and on the left, ii) a zoomed-in view of the 5^th^ order peak shows (schematically) the shift of the peak-centre (red circle) to larger wavevector ((c): white arrow) on loading (UL → L). (e) and (f) show 1D integrated I(q) and I(χ) plots corresponding to the schematic 2D patterns in (c) and (d), showing the peak shift and broadening in I(q) and the peak intensity reduction and broadening in I(χ). (g) and (h) model of the nano- and microstructural dynamics of cartilage collagen fibrils under cyclic compression, showing (g) reduction of fibril pre-strain (at a fixed orientation) and (h) top: cylindrically/axially symmetric broadening of the fibril orientation distribution; in (h) a color-coding is used to depict the fibril angle with respect to sample (and loading) axis: red–close to vertically oriented fibrils, at 90° to the joint surface; green–off-axis fibrils relative to the joint surface. (h): bottom–load-induced fibril orientation change wI_χ_ is reduced in IL-1β compared to controls. Circle with arrow-heads represents the loading/unloading protocol.

To highlight the main findings, under cyclic compression the collagen fibrils exhibit a reversible reduction in the fibrillar D-period characterizing the pre-strain, in both control- and inflammatory- groups (Fig.3 and Supplementary **Fig. S**4). The reduction is likely due to pressure-induced reduction in localized matrix hydration, which will reduce the hydrostatic swelling pressure from the matrix which contributes to fibrillar pre-strain [6,[50], [51], [52]]. This mechanism is indicated in the schematic in Fig. 5**(e) and (g)**, whereby under compression there is a shift in the azimuthally integrated peak *I*(q). Though a similar fibrillar response has been shown by us previously in cartilage cores under static compression and quasi-static loading [12], here we show that the recoverable nature of the phenomenon at the intra-fibrillar level under dynamic (not static) loading, which is likely driven by the re-hydration of the cartilage as water is drawn back into the intrafibrillar space [51]. Further, there is an indication that over successive cycles the total difference in the D-period between loading and unloading begins to reduce, suggesting that as the tissue tends towards an equilibrium state over repeated loading–which has been previously supported in a non-linear poroelastic model [53]–the fibrils respond accordingly. While the D-period changes are small (∼0.2-0.3 nm or ∼0.3%), the magnitude is similar to load-induced fibrillar-level strains in other collagenous tissues (e.g., tendon [54,55] or cornea [56]) and consistent with our prior work on AC static compression and stress relaxation [11,12]. Due to the high elastic modulus of collagen fibrils (∼0.5 GPa [57]), these small fibril strains correspond to significant stress levels (∼MPa) in the tissue, comparable to the maximum stresses in stress relaxation in bovine cartilage [12].

In parallel to the D-period changes, the inter-fibrillar axial disorder (parameterized by w_q_) significantly increases upon compression in both conditions as indicated in Fig. 3**(d–f)** and Supplementary **Fig. S**4 (e–h). This suggests that as fluid is pushed out from the inter-fibrillar space under loading [45,58], the range and variability in D-periodicity varies across the fibrillar network, which could indicate that individual fibrils experience variable levels of fluid-induced pre-strain across both their individual lengths as well as between fibrils. This variation in pre-strain (via D-period) leads to a broadening of the azimuthally integrated peak, coupled with a reduction in the peak height. However, if the sole mechanism were an increase in inter-fibrillar variability in D-period for fibrils in-plane (satisfying the SAXS diffraction condition), the total intensity of each peak (intensity ∝ peak height × peak width) would remain constant. Experimentally, though, the I_5_ peak intensity indicated in Fig. 3**(g–i)** shows a significant reduction in intensity under compression. This effect can be attributed to changes in the angular distribution of the fibrils in 3D, with fibrils moving out of the 2D detector plane, as we will now show.

When considering the angular orientation of the fibrillar network, it appears that the mean direction of orientation is nearly unchanged on compression (∼2-6% under loading; Fig. 2**(a–c)**). This may be due to the choice of strain control rather than load control as a previous study Moger *et al.* showed that cartilage reorientation only occurred under surface pressures above 2.6 MPa [40]. However, it is also important to distinguish between the direction of orientation (the peak position of the radially integrated intensity I(χ)) and the degree of orientation (related to the width of the peak shown in Fig. 2**(g)**). There is a clear compression-induced increase in the 2D angular span over which the fibrils are oriented, as indicated by the broadening of the I(χ) peak. Further, this increase is recoverable back to its original configuration as indicated in Fig. 2**(d)**.

The foregoing results, particularly the increase in wI_χ_ and decrease in I_5_ upon compression, can be understood via a 3D interpretation of the SAXS signal in terms of the ECM deformation mechanisms underpinning this change (Fig. 5). First, we note that the SAXS beam going through the macroscopic tissue core is probing fibrils at all possible orientations around the vertical axis–i.e. with fibre symmetry around the cylindrical sample axis. This is because of the well-known arcade-like Benninghof fibril motif in articular cartilage [59,60], along the occurrence of this motif, rotated around the vertical axis, along the beam path. As a result, the overall fibril distribution has a conically symmetric distribution as shown in Fig. 5**(h)**. In reciprocal space, the original conical fibril distribution leads to a SAXS meridional scattering intensity consisting of 3D conical-surfaced ellipsoids (Fig. 5**(b)**, left), whose opening angle is proportional to the width of the real-space 3D conical distribution. The intersection of this 3D distribution with the (flat for SAXS) Ewald sphere leads to a series of meridional arcs, corresponding to the measured patterns [61]. Under compression, a propagation of tissue-level deformation leads to a zone-dependent strain in the tissue, as previously reported [62], [63], [64]. In the deep zone, a vertical compression will be expected to broaden the conical fibril distribution, as also seen in static loading [40,41]. In parallel, in reciprocal space on compression (Fig. 5**(b)**, right), the 3D opening angle of the conical ellipsoids will increase with the broadening of the real-space distribution (Fig. 5**(h)**, right). As there is no addition or removal of fibrils during deformation, the integrated SAXS intensity in the 3D ellipsoid is conserved. Therefore, it can be easily shown that the total intensity of any 2D SAXS slice will reduce, to counterbalance the 3D broadening effect. By coding this simple SAXS model in a 3D graphical representation (Fig. 5**(b–f, h)**), it is thus seen that the effect of an axially symmetric 3D broadening of the fibril distribution is exactly the reduction of total peak intensity (from I(χ)) and increase in wI_χ_ seen experimentally. On removal of load, as with the changes to the molecular organization, the angular reversibility back to the original configuration is likely to be driven by the PG and associated hydration that together act in a gel-like manner to drive the fibre-composite like structure to retain its original shape [17,65,66]. Further refinement to this physical picture may consider water-induced changes in relative scattering contrast between the fibrils and extrafibrillar matrix, but the magnitude of the 3D broadening appears sufficient for the main effect,

When considering the effects of IL- 1β driven inflammatory conditions, we show that though there are changes to the macroscale level mechanics, the fibrils still have the capacity to reversibly reorder at the supramolecular level in the same manner as the control samples. The D-period is reduced on compression and recovered (Supplementary Fig. S**4**), but the total difference (and significance of these values) between the loaded and unloaded values reduces faster in the IL-1β group, as shown by a reduction in the number of + signs after 60 cycles (Supplementary **Fig. S4(c)**). This trend also applies in the total difference in w_q_ (inter-fibrillar axial disordering), where the difference becomes less apparent in successive cycles. Such an observation would indicate that there is less intra-sample variability in the fibrillar D-periodicity in IL-1β- vs. controls, which could be due to a reduction in the localised swelling pressure as a result of the matrix degradation associated with IL-1β treatment. IL-1 is known to induce a pro-inflammatory cascade, leading to the release of MMPs causing cartilage degradation [35]. The reduced difference between the loaded- and unloaded states indicates a reduction in flexibility or extensibility of the fibrils under inflammatory conditions, perhaps related to matrix- and hydration-changes [31,67]. However, it is noted that the superficial zone of AC is affected most by IL-1 treatment, with Li et al [68] showing minor collagen loss in the bulk tissue, while here the deep zone was probed with the X-ray beam (due to constraints imposed by the X-ray method). Depth-dependent variation in cytokine response to IL-1 may be caused by a variety of factors including epitope availability and penetration of the cytokine. Nevertheless, there is evidence (from our SAXS analysis) of some collagen loss or degeneration in the deep zone, with Supplementary **Fig. S5(a)** showing a small (∼10–15%) reduction in I_5_ peak intensity between control and IL-1β samples.

The fibrillar direction of orientation remains relatively unchanged in the inflammatory group, but the main difference in angular response in the fibrils in the IL-1β group relative to control is the inability for the fibrillar network to spread out under compression. Supplementary **Fig. S3(h)** shows that the fibrils show no significant difference in the degree of orientation, and the total difference between loaded and unloaded in wI_χ_ is also significantly less compared to control (Supplementary **Fig. S3(g)**). As we interpret changes in I(χ) as linked to alterations in the 3D distribution and showing up in changes in total intensity I_χ_ and wI_χ_, the reduced significance of these differences (Supplementary **Fig. S5**) may relate to the reduced flexibility in the fibrillar network to change configuration under compression.

A limitation in our work is the number of cycles that the explants were exposed to and the reflection of what these findings represent in terms of joint level mechanisms. The hydration driven changes to the deformation mechanisms of cartilage has previously been modelled under cyclic loading by Zhang et al [53], who have developed a biphasic, large deformation, non-linear poroelastic model. They found that under steady state cyclic loading, the pore fluid exuded from the tissue was exactly equal to the pore fluid imbibed by the tissue during each cycle beyond around 500 cycles [53]. Combining this knowledge with our findings, this would suggest our measurements reflect more the initial equilibrium of the tissue under cyclic loading for example when a joint is first loaded rather than the longer-term effects. This would suggest that over longer-term successive cycles the internal hydration of the explants remains relatively unchanged, and therefore the matrix level changes we observe may also equilibrate over time. To develop our understanding further, the explants will be studied under a higher frequency of loading but also develop a load-controlled configuration to represent a more physiological response–which may also drive more observable differences in the inflammatory treatment group. Further it would be beneficial to observe such changes in human cartilage where the depth wise fluid flow and deformation mechanisms are considered given the strain propagation variability throughout the different zones of cartilage [69,70].

In conclusion, we have applied a bespoke quantitative nanoscale imaging approach to reveal the fibrillar level mechanisms that underpin the tissue level mechanics under cyclically loaded cartilage. Combining the observed reversible reduction in fibril pre-strain (D-period) with the change in degree of orientation, it is apparent that under compression the fibrillar network in cartilage plays a significant role in resisting load through the reorganization both at the inter-fibrillar (via 3D reorientation and pre-strain variability) and fibrillar (via fibril pre-strain reduction) levels. Further, we show that under inflammatory conditions following IL-1β treatment, while there are insignificant changes to the predominant response of the molecular level mechanisms under repetitive compression, the flexibility of the fibrillar network and ability to broaden the degree of orientation is reduced and this becomes more apparent longer term which reflect the macroscopic poroelastic changes. Going forward, understanding how this baseline pattern of nanoscale deformation is altered in degenerative disorders like osteoarthritis and in ageing, and–in particular–in human tissue, will provide important clues as to the matrix-level changes which alter the mechanobiology of cartilage and influence disease progression.

## Declaration of Competing Interest

The authors declare that they have no known competing financial interests or personal relationships that could have appeared to influence the work reported in this paper.

## Appendix Supplementary materials

Image, application 1
