# Supplementary material for: Reversible changes in the 3D collagen fibril architecture during cyclic loading of healthy and degraded cartilage
Source: Acta Biomater. 2021 Dec;136:314–26. doi: 10.1016/j.actbio.2021.09.037 (PMC8631461; doi:10.1016/j.actbio.2021.09.037)
Supplement: Supplementary file 1 [file mmc1.docx]

**Supplementary Information:**

Schematic of fibrillar structure and relation to SAXD pattern:


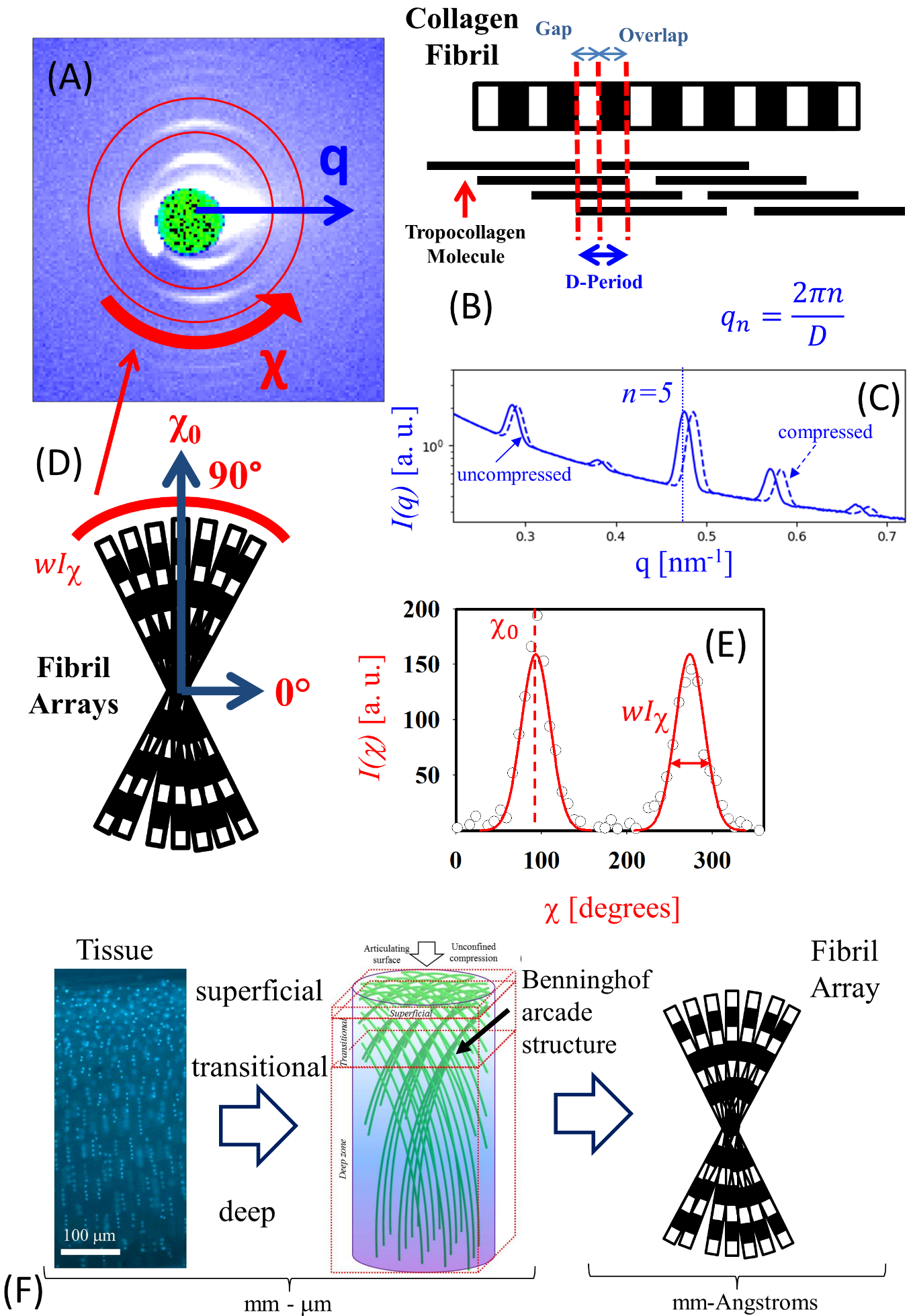


**Figure S1: *Relation of 2D SAXD pattern to fibrillar ultrastructure.* (a) Representative 2D SAXD pattern showing the arced peaks from meridional collagen D-periodicity. The radial (q) and azimuthal (χ) directions are shown on the pattern. (b) Schematic of the staggered tropocollagen molecular arrangement inside the fibrils (bottom) leading to the D-period ~65-67 nm, leading to a axially periodic electron density pattern at the level of the fibril (top) with dense (overlap: dark) and less dense (gap: white) regions. (c) Schematic of azimuthally averaged 1D intensity profiles as a function of radial wavevector q in unloaded (solid) and loaded (dashed) states. The 5^th^ order peak is at q_05_ = 5 × 2π/D. (d) In the tissue, arrays of fibrils at different angles lead to a distribution of the SAXD intensity in the azimuthal (χ) angular direction. (e) Example of radially averaged azimuthal intensity plot I(χ) showing peaks along the main direction of fibril orientation χ_0_ and the width of the angular distribution w_χ_. (f) Schematic of the tissue structure at the mm-μm scale (left: epifluorescence image) showing the Benninghof arcade like orientation of fibres (centre; schematic) where the individual fibres are comprised of fibril arrays (right).**

Influence of IL-1β on tissue level mechanics


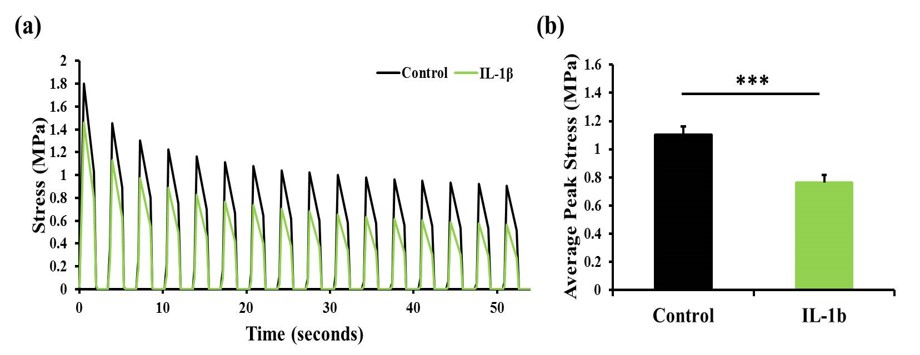


**Figure S2: *IL-1β treatment leads to a reduction in the tissue level stress under 20% strain in cyclic loading in bovine explants.* (a) Stress measured over the first 16 cycles, Black line indicates the control samples (n=7) that were kept in media whilst the green line indicates the samples treated with 5ng/ml of IL-1β in media (n=6), (b) average peak stress from first 16 cycles. Error bars represent standard error of mean and * refers to significance between the groups (p<0.01).**

Influence of IL-1β on the angular distribution of the fibrils

There is a minimal difference between the two treatment groups in the predominant orientation angle of the fibrils in both the unloaded and loaded case (**Figure S3** (a) and (b)), suggesting that IL-1β treatment does not affect the orientation configuration of the fibrillar groups. When considering the total difference in the orientation between the loading phases in **Figure S3** (c), the control and IL-1β groups shown a similar trend in the changes to the direction of orientation over successive cycles. Similar to the controls, this trend is not significant for IL-1β, as seen in **Figure S3** (d), where the average of each sample over the full range of cycles remains unchanged between loading and unloading (as with the control group in **Figure** **1**).

**
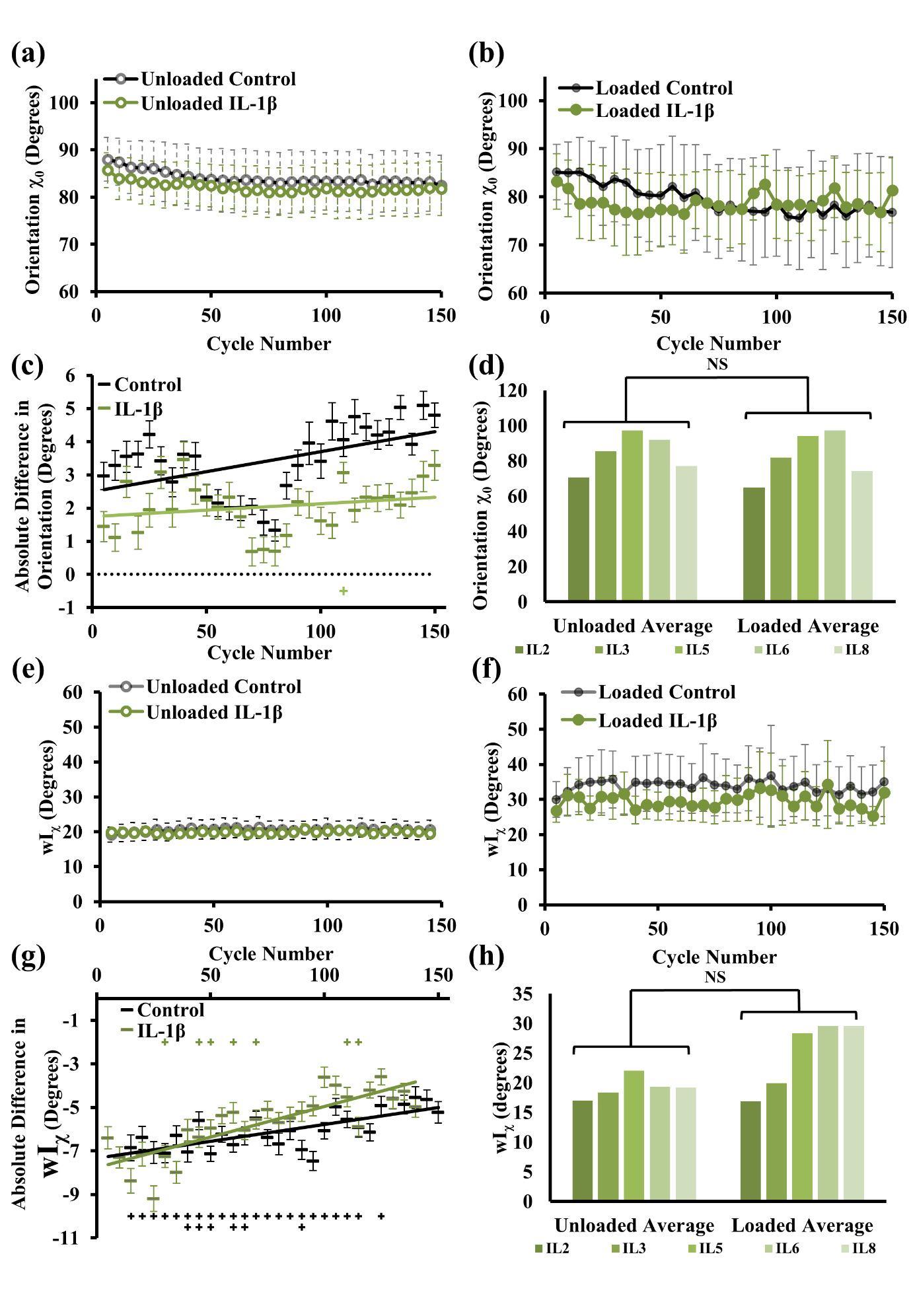
**

**Figure S3: IL-1β treatment leads to an inability of the fibrils to significantly change their angular degree of orientation under compression, accompanied by no change to the predominant orientation direction of the fibrils. The long-term cyclic trend in both the unloaded and loaded phase of each cycle for fibrillar orientation and the degree of orientation is shown in (a)-(b) and (e)-(f) respectively where the grey points represent the control group whilst the green represents the IL-1β group. The absolute pairwise difference in the two phases for each sample group for the orientation and degree of orientation is shown in (c) and (g) respectively, where the black points represent the control samples whilst green the Il-1β treatment group. In both, the two lines are linear regressions as a guide to the eye and the dashed line indicated 0 where applicable. The (+) symbols indicate the significant difference of the unloaded and loaded phase at each point where black (+) represents the control group points and green (+) represent the IL-1β group points. The average of the long-term trends for each sample within each phase for the orientation and degree of orientation is shown in (d) and (h) respectively, where there is no significant change in both parameters under cyclic loading. Error bars represent standard error of mean throughout where n=5. The + symbol in parts (e) and (g) indicates the significance of the difference between the unloaded and loaded phase at each measured cycle, where p<0.05 (+) and p<0.01 (++).**

Influence of IL-1β on the D-periodicity and associated parameters

**Figure S4** (a) shows the trends in bovine cartilage explants treated with 5ng/ml of IL-1β. In the uncompressed state, the D-period is lower across the full cycle range for the IL-1β group, compared to controls, by around 0.12%, a value which is less than the pre-strain reduction observed for chondroitinase ABC treatment (at around 1.1%), in our prior work on similar bovine cartilage explants [1]. The difference between IL-1β- and control groups is then less apparent under loading, as shown in **Figure S4** (b), where at the start of the cyclic loading there is a small difference, but then the two groups begin to overlap from 50 cycles onwards. As with the control (**Figure S3(a-c)**), there is a clear initial reduction in D-period under compression, followed by a recovery in the IL-1β treatment group.

**
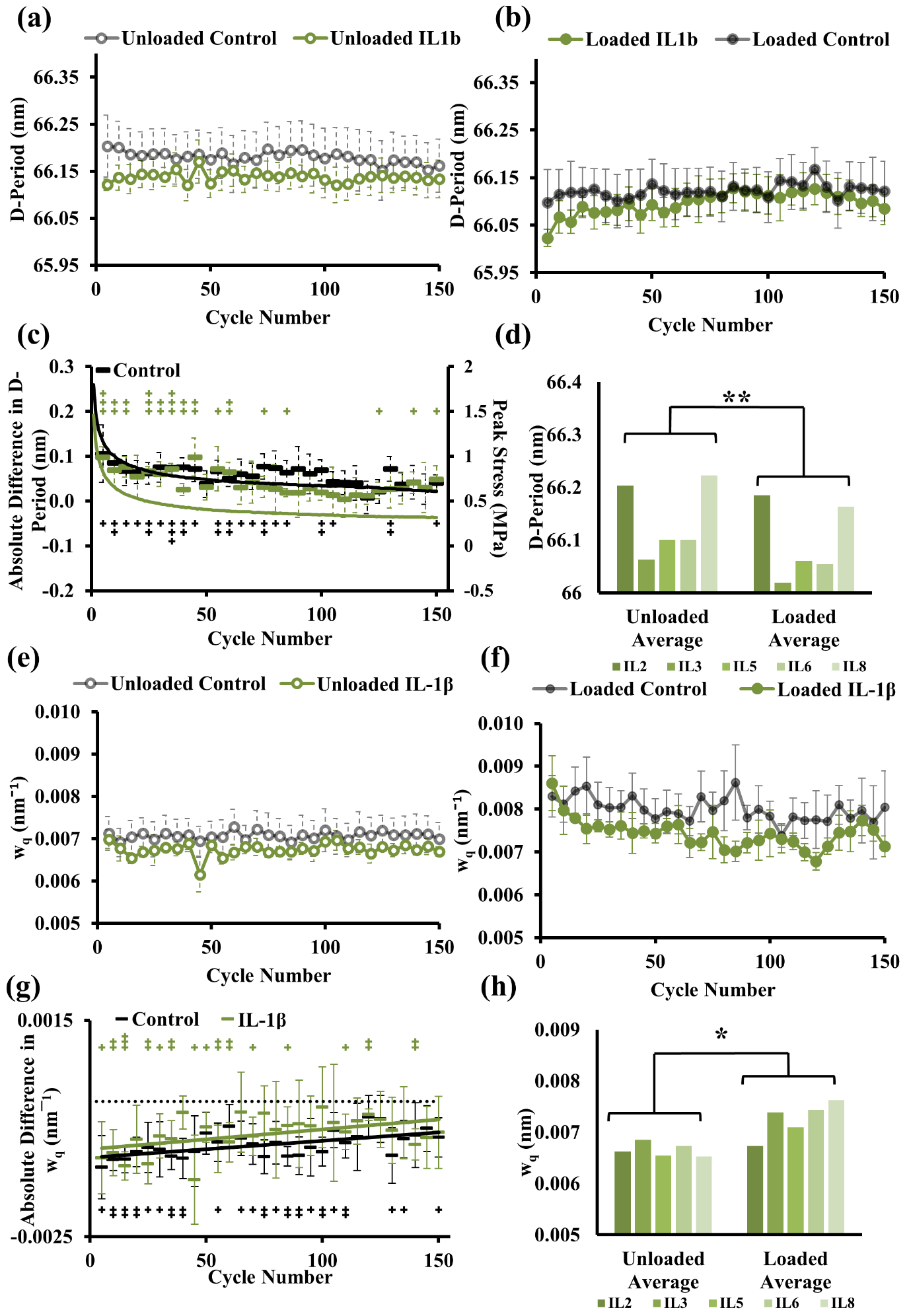
**

**Figure S4: *While IL-1β treatment causes a macroscale level change to the mechanics, the fibrils still have the ability to reversibly change pre-strain and interfibrillar heterogeneity at the supramolecular level*. The long-term trend in D-period and w_q_ when unloaded and loaded are shown in (a)-(b) and (e)-(f) respectively, where the grey points represent the control average whilst the green represents samples treated with 5ng/ml of IL-1β in each loading phase. The absolute pairwise difference in the two phases for each sample group for the D-period and w_q_ is shown in (c) and (g) respectively, where the black points represent the control samples whilst green the Il-1β treatment group. In part (c), the two corresponding trend lines show the averaged peak stress over successive cycles as read by the right axis, whilst in part (g) the two lines are linear regressions as a guide to the eye. The (+) symbols indicate the significant difference of the unloaded and loaded phase at each point where black (+) represents the control group points and green (+) represent the IL-1β group points. As with the control group in Figure 3, there is a significant reduction in D-period under cyclic load, as shown in (d) where the long-term average is taken for each sample. The reduction is accompanied by a similar increase in the inter-molecular disordering under load, as indicated by the w_q_ parameter in (h). Error bars represent standard error of mean throughout where n=5, and * indicates the significance between the loaded and unloaded groups, where p<0.05 (*) and p<0.01 (**). The + symbol in parts (b), (e) and (h) indicates the significance of the difference between the unloaded and loaded phase at each measured cycle, where p<0.05 (+), p<0.01 (++) and p<0.001 (+++).**

Influence of IL-1β on the I_5_ peak intensity

**
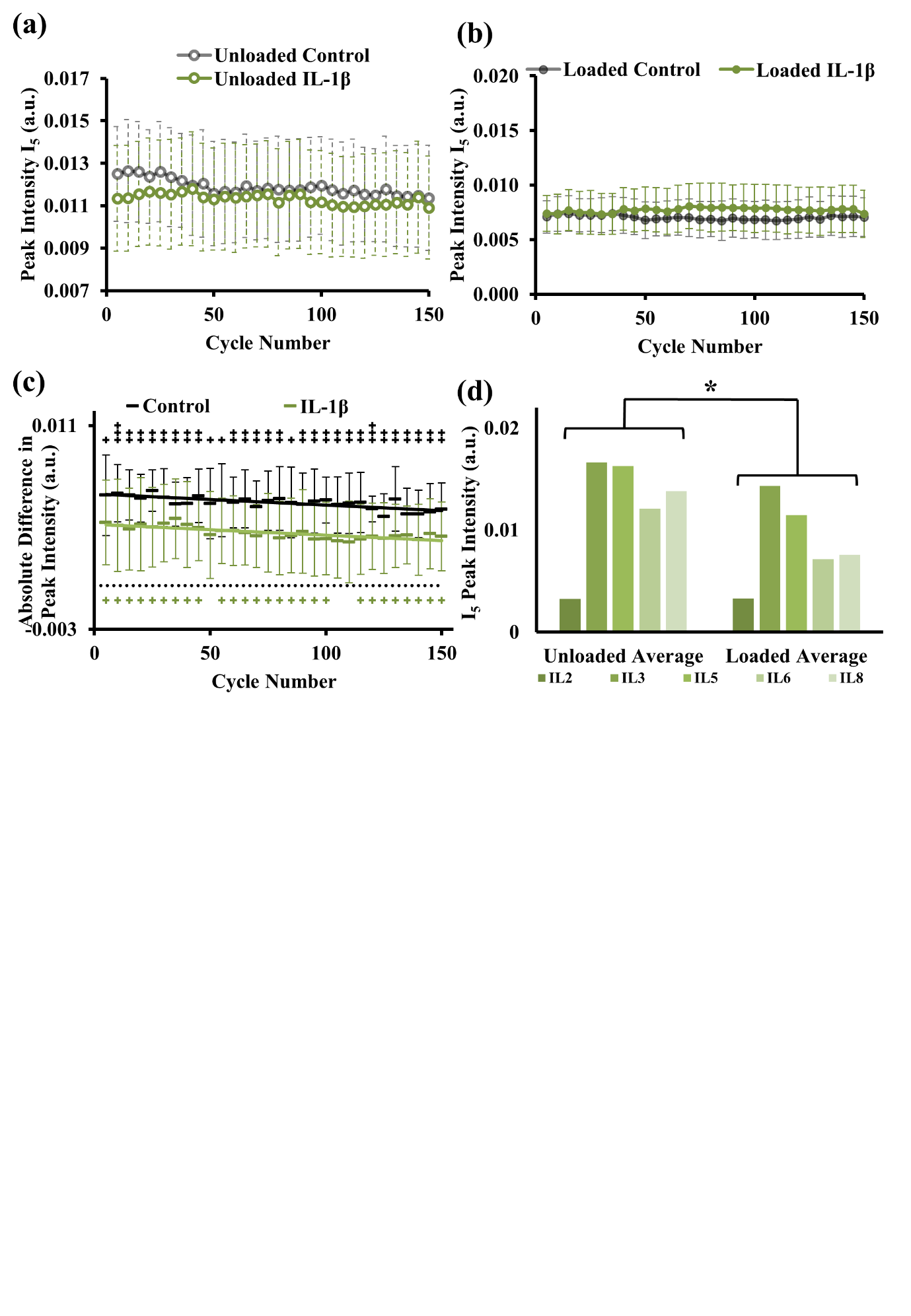
**

**Figure S5: A reduction in peak intensity is observed in the IL-1β group, but the difference is less apparent when compared to the control group. The long-term cyclic trend in both the unloaded and loaded phase of each cycle is shown in (a) and (b) respectively, where the grey points represent the control group whilst the green represents the IL-1β group. The absolute pairwise difference in the two phases for each sample group for the peak intensity is shown in (c), where the black points represent the control samples whilst green the Il-1β treatment group. Here, the two lines are linear regressions as a guide to the eye and the dashed line indicates a zero value. The (+) symbols indicate the significant difference of the unloaded and loaded phase at each point where black (+) represents the control group points and green (+) represent the IL-1β group points. The average of the long-term trends for each sample within each phase is shown in (d), where a significant reduction in peak intensity can be observed. Error bars represent standard error of mean throughout where n=5, and * indicates the significance between the unloaded and loaded groups, where p<0.05 (*) and p<0.01 (**). The + symbol in parts (c) indicates the significance of the difference between the unloaded and loaded phase at each measured cycle, where p<0.05 (+), p<0.01 (++) and p<0.001 (+++).**

**Table of Abbreviations**:

| **Name** | **Abbreviation** |
| --- | --- |
| Articular Cartilage | AC |
| Small angle X-ray scattering | SAXS |
| Interleukin 1-beta | IL-1β |
| Extracellular matrix | ECM |
| Matrix metalloproteinase | MMP |
| Phosphate buffered saline | PBS |
| Full width at half maximum | FWHM |
| Transistor-transistor logic | TTL |
| Axial width of meridional SAXS peak | w_q_ |
| Azimuthal width of meridional SAXS peak | wI_χ_ |

**Supplementary Information References**:

[1] S.R. Inamdar, D.P. Knight, N.J. Terrill, A. Karunaratne, F. Cacho-Nerin, M.M. Knight, H.S. Gupta, The Secret Life of Collagen: Temporal Changes in Nanoscale Fibrillar Pre-Strain and Molecular Organization during Physiological Loading of Cartilage, ACS Nano. 11 (2017) 9728–9737. https://doi.org/10.1021/acsnano.7b00563.
